# Unlocking Roadside Carbon Sequestration Potential: Machine Learning Estimation of AGB in Highway Vegetation Belts Using GF-2 High-Resolution Imagery

**DOI:** 10.3390/s26051729

**Published:** 2026-03-09

**Authors:** Weiwei Jiang, Heng Tu, Qin Wang

**Affiliations:** School of Civil Engineering, Architecture and Environment, Hubei University of Technology, Wuhan 430068, China; 102311044@hbut.edu.cn (H.T.); 20140084@hbut.edu.cn (Q.W.)

**Keywords:** aboveground biomass, remote sensing variables, machine learning, random forest, GF-2

## Abstract

Aboveground biomass (AGB) is a key indicator of vegetation productivity and terrestrial carbon stocks; therefore, robust AGB estimation is critical for assessing ecosystem services and carbon cycle research. Previous studies have largely focused on forest and cropland ecosystems. In contrast, roadside vegetation along highways and other linear transport corridors remains comparatively underexplored despite its potentially important role as a carbon sink. Here, we integrate field-measured AGB samples with GF-2 high-resolution satellite imagery to evaluate the suitability of multiple remote-sensing predictors and machine-learning algorithms for estimating AGB in highway roadside vegetation. Six remote-sensing variables were used as predictors, including four vegetation indices (Normalized Difference Vegetation Index (NDVI), Perpendicular Vegetation Index (PVI), Enhanced Vegetation Index (EVI), and Modified Soil-Adjusted Vegetation Index (MSAVI) and two-band ratios (B342 and B12/34). Five regression models—multiple linear regression (MLR), partial least squares regression (PLSR), random forest (RF), support vector regression (SVR), and extreme gradient boosting (XGBoost)—were developed and systematically compared under both single-variable and multi-variable scenarios. Model performance was evaluated using five-fold cross-validation, with the coefficient of determination (R^2^) and the root mean square error (RMSE) as metrics of evaluation. The results indicate that the RF model under the multi-variable scenario achieved the best overall performance, with a training R^2^ of 0.83 and a testing RMSE of 0.84 kg·m^−2^, substantially outperforming the other linear and non-linear models. The optimal RF model was further applied to GF-2 imagery to produce a spatially explicit AGB map for a 32 km highway segment and a 30 m roadside buffer on both sides, yielding an estimated total aboveground biomass of 566.97 t for the corridor. These findings demonstrate that combining high-resolution remote sensing with machine-learning approaches can effectively improve AGB estimation for linear roadside vegetation systems, providing technical support for ecological monitoring, roadside greening management, and carbon accounting for transport infrastructure.

## 1. Introduction

One of the primary drivers of global climate change is the accumulation of greenhouse gases, with carbon dioxide (CO_2_) playing a particularly critical role [1]. This has intensified the need for effective strategies that both reduce emissions and enhance carbon sequestration. Terrestrial ecosystems, especially forests and woody vegetation, act as major carbon sinks and play a vital role in offsetting anthropogenic emissions [2,3]. In this context, accurately quantifying carbon stocks in terrestrial ecosystems is essential. Aboveground biomass (AGB), a core indicator of vegetation productivity and carbon storage, exerts a strong influence on the global carbon cycle and climate regulation [4]. Reliable AGB estimation is therefore indispensable not only for fundamental ecological research, but also as a scientific basis for climate-change mitigation and sustainable land-management decision-making.

Rapid urban expansion and the accelerating development of transportation networks have given rise to distinctive linear roadside ecosystems. Although roadside vegetation belts are typically narrow, their cumulative area across national and global road networks is substantial. These vegetation strips deliver multiple ecosystem services, including air purification, noise attenuation, biodiversity support, and meaningful carbon storage [5]. However, compared with well-studied ecosystems such as forests and croplands, the carbon-sink capacity of roadside vegetation—and its robust quantification—has received far less scientific attention [6]. This knowledge gap constrains the integration of roadside ecosystems into regional carbon accounting frameworks and sustainable development planning.

Remote sensing offers a cost-effective means of monitoring AGB from regional to global scales [7]. Conventional approaches commonly rely on moderate-to-coarse-resolution satellite imagery (e.g., Sentinel-2 and Landsat; 10–30 m pixels) to derive vegetation indices (e.g., NDVI, EVI, and SAVI), which are then linked to AGB using statistical or machine-learning models [8,9]. However, applying these approaches to linear highway vegetation presents fundamental challenges. Roadside environments are spatially heterogeneous, comprising pavement, shoulders, vegetation, slopes, and adjacent land uses, and moderate-/coarse-resolution pixels often fail to delineate narrow vegetation belts and their boundaries, leading to severe spectral mixing. Moreover, roadside vegetation frequently exhibits strong within-belt gradients and fragmentation (e.g., due to species composition, management intensity, and disturbance). Because the width of vegetation strips is often comparable to—or smaller than—the pixel size of moderate-resolution sensors, fine-scale structural information is lost, substantially limiting AGB retrieval accuracy [8,10,11,12].

To address these constraints, we use sub-metre multispectral imagery from China’s Gaofen-2 (GF-2) satellite (0.8 m spatial resolution). This spatial detail effectively reduces mixed-pixel effects in roadside settings and enables the precise characterization of belt boundaries, internal structural heterogeneity, fragmentation, and micro-scale gradients [10,13]. We pair these data with advanced machine-learning algorithms (e.g., random forest, gradient boosting, and support vector regression) to estimate AGB.

Our framework captures potentially complex, non-linear relationships between AGB and high-resolution spectral features, such as multispectral reflectance and derived vegetation indices or remote-sensing variables. Specifically, our objectives are to: (1) systematically evaluate the predictive performance and robustness of several machine-learning models for estimating AGB in linear highway vegetation belts using GF-2 imagery and multi-source remote-sensing predictors; (2) quantify the contributions of different remote-sensing variables (spectral bands, vegetation indices, and derived predictors) and identify key features driving AGB estimation; (3) apply the optimal model to create a spatially explicit AGB map for the highway network within the study area to support fine-scale management; and (4) assess the spatial patterns of AGB and the cumulative carbon-stock potential of linear vegetation belts, thereby examining their prospective contribution to regional carbon sequestration, sustainable roadside ecological management, and inclusion in national or local carbon accounting systems.

By integrating field ecological measurements, high-resolution remote sensing, and machine learning, this study provides a methodological framework for quantifying carbon stocks in complex linear ecosystems. It advances scientific understanding of roadside vegetation systems. The resulting products and insights can inform sustainable transport infrastructure planning and management [5].

## 2. Materials and Methods

### 2.1. Study Area

**Figure 1:**
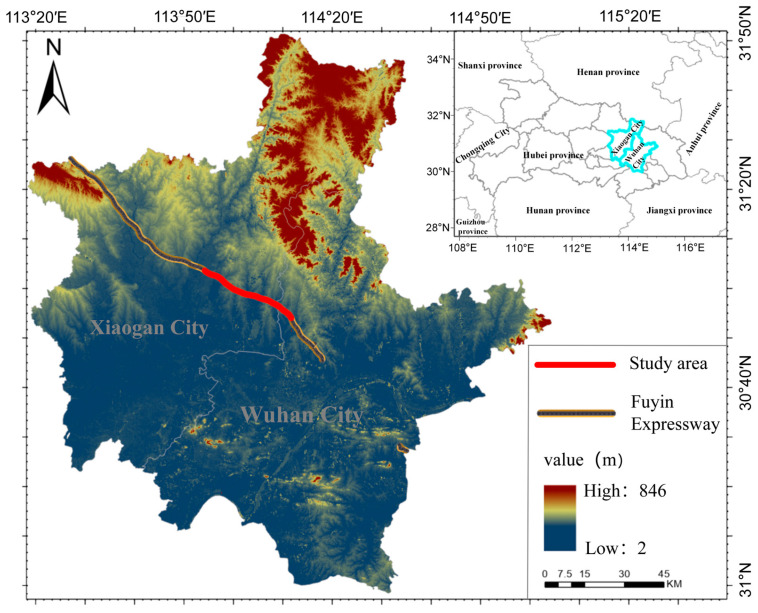
Safety risks induced by ice shedding.

The Fuzhou–Yinchuan Expressway (Fuyin Expressway), with the study area shown in Figure 1, is a major highway in China that connects Fuzhou City in Fujian Province with Yinchuan City in the Ningxia Hui Autonomous Region. It constitutes the 14th east–west trunk route within China’s National Expressway Network. The expressway begins at the Qingkou Interchange in Minhou County, Fuzhou (revised in the 2022 National Highway Network Plan to Fuzhou Changle International Airport), and terminates at the Yinchuan Interchange in Xingqing District, Yinchuan, with a total length of 2399.003 km.

The study area lies in the mid-to-low latitudes and is characterized by a humid subtropical continental monsoon climate with distinct seasons. The mean annual temperature is approximately 16 °C, the frost-free period is long, and annual precipitation ranges from 1100 to 1450 mm. The study corridor covers a 32 km highway segment with an effective analysis width of approximately 90 m, corresponding to a total area of about 2.88 km^2^. The maximum recorded wind speed is 29.6 m s^−1^; prevailing winds are predominantly northwesterly, with peak wind intensity reaching Beaufort force 10. Southerly winds occur more frequently in June and July, whereas northerly winds dominate during the remaining months. These climatic conditions are conducive to roadside vegetation growth and its potential carbon sequestration function.

### 2.2. Data Acquisition and Processing

The datasets used in this study include GF-2 satellite imagery, ground-based AGB sample plots, and auxiliary road vector data. Table 1 summarizes the source of receipt, spatial resolution, and acquisition conditions of all initial datasets.

#### 2.2.1. Field Data Acquisition and Processing

This study focused on key sections along the Xiaogan segment of the Fuyin Express way, including the Xiaogan Service Area, the Xiaochang Interchange, and other representative roadside vegetation zones. A total of 120 vegetation plots were established across both sides of the road corridor, as well as within the service area and interchange environments. Within each plot, we conducted field inventories of vegetation attributes, including species composition, abundance, diameter at breast height (DBH), plant height, crown dimensions, and canopy cover. Site photographs were taken to document landscape conditions, and the geographic coordinates of each plot were determined with centimetre-level accuracy using a Qianxun Xingyao Xplus RTK device (Qianxun Spatial Intelligence Inc., Shanghai, China); elevation was recorded simultaneously.

Aboveground biomass (AGB) of trees and shrubs was estimated using species-specific or functional-group-based allometric equations (Table 2). For dominant tree species, including *Cinnamomum camphora* and *Populus* spp., published national allometric equations were adopted. When species-specific equations for Hubei Province were unavailable, functional group equations developed for subtropical broadleaf forests were used as substitutes. These equations have been widely applied in regional biomass and carbon stock assessments under comparable climatic and ecological conditions [14,15].

For herbaceous vegetation, AGB was quantified using a direct harvest method. All aboveground plant material within each plot was clipped, oven-dried to constant mass, and subsequently weighed to obtain dry biomass.

#### 2.2.2. GF-2 Data Acquisition and Processing

We used Gaofen-2 (GF-2) satellite imagery as the primary remote-sensing data source. GF-2 is a key component of China’s High-Resolution Earth Observation System (CHEOS) and was launched on 19 August 2014. The GF-2 imagery used in this study was obtained from the China Centre for Resources Satellite Data and Application (CRESDA), which is the official data distribution agency for CHEOS. The satellite operates in a sun-synchronous orbit at an altitude of approximately 631 km, with a swath width of about 45 km and an agile pointing capability that enables a nominal revisit interval of approximately 5 days.

The GF-2 system comprises two distinct, co-aligned cameras: a panchromatic (PAN) sensor (0.8 m) and a multispectral (MUX) sensor with four bands (blue, green, red, and near-infrared) at 3.2 m resolution. All preprocessing was performed in ENVI^®^ v5.6 (NV5 Geospatial Solutions, Inc., Boulder, CO, USA) [19]. Radiometric calibration was applied to convert DN values to radiometrically calibrated products (stored in BIL format), followed by atmospheric correction using the QUAC module to derive surface reflectance for the MUX bands. Because PAN and MUX are acquired by different sensors, geometric alignment between the PAN and MUX images was ensured within the pan-sharpening workflow. The PAN image and corrected MUX image were then fused using the ENVI Toolbox task “NNDiffuse Pan Sharpening” to generate 0.8 m multispectral imagery for vegetation index/band-ratio computation and AGB mapping. All spatial products and map outputs were referenced to the WGS 84 geographic coordinate system (EPSG:4326), with latitude and longitude expressed in decimal degrees (°). The imagery was acquired in May 2023 during the local growing season under minimal cloud cover. Detailed sensor specifications are provided in Table 3.

### 2.3. Steps for AGB Estimation

**Figure 2:**
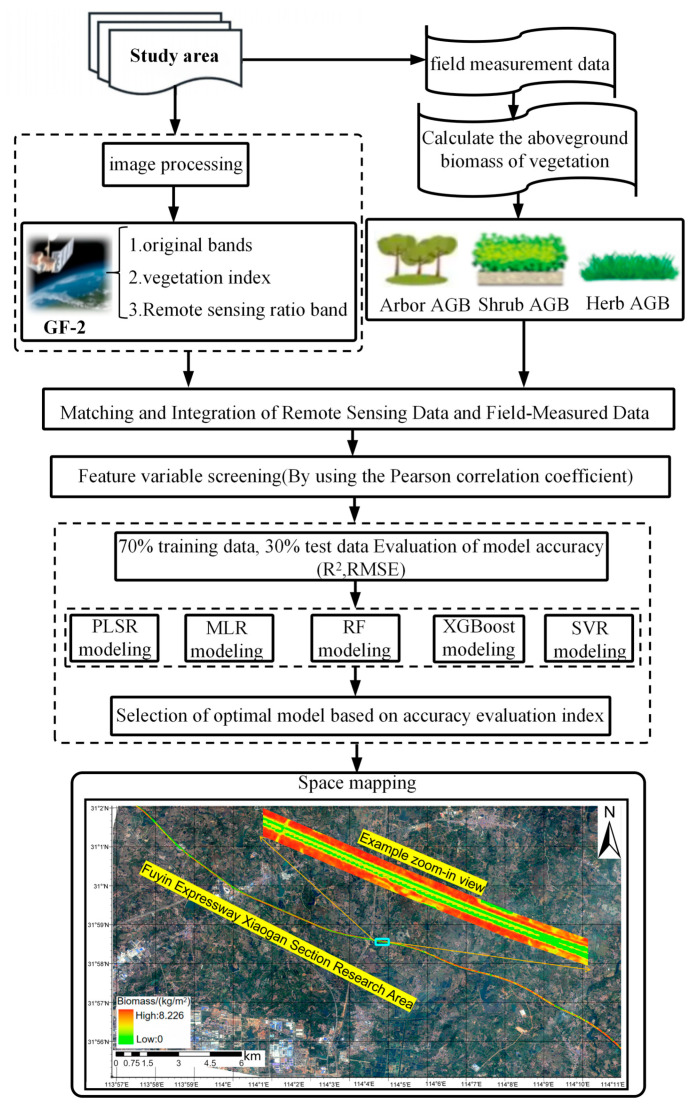
Workflow of the study.

The overall workflow of this study comprised four main steps (Figure 2): (1) data acquisition and preprocessing; (2) selection of appropriate predictor variables based on field observations and satellite data; (3) comparison of multiple machine-learning algorithms and development of an aboveground biomass (AGB) estimation model; and (4) pixel-wise application of the optimal model to satellite imagery to estimate total aboveground biomass across the roadside corridor.

#### 2.3.1. Derivation of Spectral Indices and Band Ratios

Using ArcGIS Pro 3.4 (Esri) [20], we clipped the GF-2 pan-sharpened multispectral imagery at 0.8 m resolution and delineated a 30 m buffer on both sides of the highway centerline to extract the roadside corridor imagery for analysis. To fully exploit the information content of multispectral remote sensing for vegetation monitoring, we constructed a set of candidate spectral predictors from the blue, green, red, and near-infrared bands. First, we derived band-ratio features, including two-band ratios as well as three-band and four-band ratio combinations, following the general design principle adopted in the MODIS Vegetation Index (MOD13) algorithm documentation [21], which states that multi-band combinations can enhance vegetation-signal sensitivity. Second, we computed a suite of widely used vegetation indices, including ARVI, DVI, EVI, GNDVI, NDVI, RVI, SAVI, and MSAVI. NDVI captures the contrast between red absorption and near-infrared reflectance. EVI can alleviate NDVI saturation under moderate-to-high canopy cover and improve sensitivity to canopy structural variation. MSAVI incorporates a soil-adjustment term to enhance vegetation characterization under sparse cover and exposed-soil conditions [22,23,24,25,26]. The full set of predictors and formulas is summarized in Table 4.

#### 2.3.2. Correlation Analysis of Remote Sensing Factors and Biomass

Selecting predictor variables that are strongly associated with biomass is crucial for reliable biomass estimation. Including irrelevant variables can increase computational burden and reduce model stability, whereas using too few predictors may hinder the development of an adequate model. Therefore, we conducted a Pearson correlation analysis to screen for predictors that were significantly related to biomass. The Pearson correlation coefficient was calculated as follows (Equation (1)):

(1) $$r=\frac{\sum_{i=1}^{n}\left(x_{i}-\bar{x}\right)(y_{i}-\bar{y})}{\sqrt{\sum_{i=1}^{n}{\left(x_{i}-\bar{x}\right)}^{2}}\sqrt{\sum_{i=1}^{n}{\left(y_{i}-\bar{y}\right)}^{2}}}$$

In the above formula, *x_i_* represents the *i*-th *x* variable, *y_i_* represents the *i*-th *y* variable and *x* and *y* represent the average value of the variables. The absolute value of *r* indicates the degree of correlation. When *r* is positive, it means positive correlation; when *r* is negative, it means negative correlation.

After removing outliers, 105 biomass samples were retained for correlation analysis. Pearson correlation statistics were computed in SPSS 26, yielding the correlations between tree biomass and the candidate predictors.

#### 2.3.3. Model Development and Algorithms

Machine learning can extract informative patterns from high-dimensional and complex datasets without imposing strict assumptions about data distributions or functional relationships, and it can flexibly accommodate predictors of different types and numbers [27]. To quantify the relationships between AGB and spectral predictors, we evaluated five regression approaches: multiple linear regression (MLR), partial least squares regression (PLSR), random forest (RF), support vector regression (SVR), and extreme gradient boosting (XGBoost). For each method, we developed AGB estimation models under both single-predictor (single-index) and multi-predictor (multi-index) scenarios. Model implementation, hyperparameter tuning, and cross-validation were conducted in R using the caret and tidymodels packages.

Multiple linear regression (MLR) is a fundamental supervised learning approach that models the relationship between predictors and a response using a linear function. Model parameters are estimated by minimizing the discrepancy between predicted and observed values, typically via least-squares optimization.

Partial least squares regression (PLSR) is a supervised method designed for high-dimensional data and multicollinearity. It performs simultaneous dimension reduction and regression by extracting latent variables that capture the shared variance structure between the predictor matrix and the response variable, conceptually analogous to principal component regression but optimized with respect to the response.

Hyperparameter tuning and model validation were performed in R software (version 4.3.2; R Foundation for Statistical Computing, Vienna, Austria) using caret and tidymodels. Hyperparameters were selected via grid search with cross-validation on the training set, and the final configuration was chosen based on the lowest cross-validated RMSE (with R^2^ and MAE used as complementary metrics). We used 10-fold cross-validation for SVR and XGBoost, and five-fold cross-validation for RF to balance estimation stability and computational cost given the different training times of these algorithms. After tuning, models were refit using the optimal hyperparameters and evaluated on an independent test set (not used during tuning) to assess generalization.

For SVR, we used a radial basis function (RBF) kernel. Hyperparameters were selected via grid search with 10-fold cross-validation, yielding an optimal cost parameter (C = 5) and RBF kernel parameter (gamma = 0.2, as implemented in e1071). Model fitting was implemented in R using a caret package with the e1071 engine.

For XGBoost, hyperparameters were tuned using grid search with 10-fold cross-validation, resulting in min_child_weight = 8, max_depth = 3, nrounds (boosting iterations) = 3000, gamma = 0.3, and reg_alpha = 0.2; the model was implemented with the xgboost package in R.

For RF, three key parameters were tuned—number of trees (ntree), number of variables tried at each split (mtry), and minimum node size (nodesize)—using grid search with five-fold cross-validation. The optimal configuration was ntree = 800, mtry = 8, and nodesize = 5, and RF modelling was performed in R using the randomForest package.

#### 2.3.4. Model Performance Assessment

To ensure a comparable assessment of generalization performance, all experiments adopted a consistent data-splitting strategy (training:test = 7:3; random seed = 42). Five-fold c-content and collinearity among predictors, thereby providing a robust evaluation of model performance for AGB retrieval.

(2) $$R^{2}=\frac{\sum_{i=1}^{n}{\left({\hat{y}}_{i}-\bar{y}\right)}^{2}}{\sum_{i=1}^{n}{\left(y_{i}-\bar{y}\right)}^{2}}$$

(3) $$RMSE=\sqrt{\frac{1}{n}\sum_{i=1}^{n}{\left(y_{i}-{\hat{y}}_{i}\right)}^{2}}$$

where *y_i_* is the observed value, ${\hat{y}}_{i}$ is the predicted value, $\bar{y}$ is the mean of the observations, and n is the sample size. R^2^ quantifies the proportion of variance in the observations explained by the model, with higher R^2^ indicating better model performance. RMSE quantifies the average magnitude of prediction errors in the same units as the response variable. Lower RMSE indicates smaller discrepancies between predicted and observed values and therefore higher predictive accuracy.

## 3. Results

### 3.1. Correlation Analysis and Selection of Remote-Sensing Predictors

**Figure 3:**
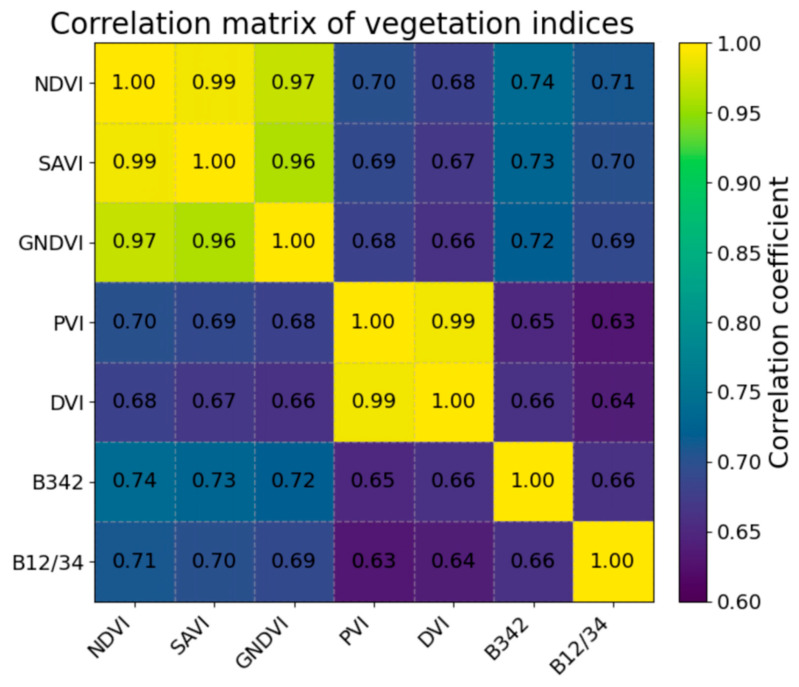
Correlation matrix of vegetation indices.

Based on the Pearson correlation analysis of the plot-level observations (Table 5), AGB was significantly and positively correlated with NDVI, GNDVI, SAVI, PVI, DVI, and EVI (r = 0.672, 0.645, 0.672, 0.650, 0.650, and 0.640, respectively; *p* < 0.001). However, the correlation matrix revealed two highly collinear clusters—{NDVI, SAVI, GNDVI} and {PVI, DVI}—with inter-index correlations reaching 0.997 (Figure 3). This indicates substantial redundancy between NDVI and SAVI, as well as between PVI and DVI. We therefore adopted a feature-selection strategy of “representative within-cluster selection and complementary across-cluster inclusion” to define the modelling predictors: NDVI was retained from {NDVI, SAVI, GNDVI}, and PVI was retained from {PVI, DVI}; EVI was additionally introduced to alleviate saturation effects [28]. In addition, B342 and B12/34 were selected because they are significantly correlated with AGB while being less redundant with the dominant red/NIR information, thereby reducing overall multicollinearity. Mechanistically, this feature set captures complementary information related to both the red–NIR contrast and key sources of confounding: NDVI (and related difference-based indices) reflects first-order red–NIR contrast, MSAVI suppresses soil-background effects under sparse vegetation cover and exposed soil conditions, EVI mitigates red-band saturation at moderate-to-high canopy cover and enhances sensitivity to canopy structure, and PVI further corrects background variability via the soil line [24,25,26,29].

In summary, six predictors—NDVI, PVI, EVI, MSAVI, B342, and B12/34—were retained as the final explanatory variables for model development. Predictor selection followed a transparent and reproducible screening procedure. First, candidate indices and band ratios were evaluated using correlation analysis and redundancy diagnostics to exclude highly collinear predictors. Second, the retained predictors were compared under identical modelling and validation settings, with performance assessed using cross-validation and an independent test set. This rule-based workflow ensures a clearly defined selection path that can be replicated across datasets.

To further evaluate the stability of the selected predictors, recursive feature elimination (RFE) was conducted using the random forest (RF) algorithm within cross-validation. This analysis was implemented as a robustness check rather than as the primary feature-selection framework, since the objective of this study is to compare single- and multi-predictor scenarios across multiple algorithms under a consistent predictor set. Given that feature-selection results are model-dependent, applying RFE separately to each regression algorithm could produce different predictor subsets and reduce cross-model comparability. The RF–RFE results are presented in the Supplementary Materials.

### 3.2. Comparison of Single-Predictor and Multi-Predictor Model Performance

**Figure 4:**
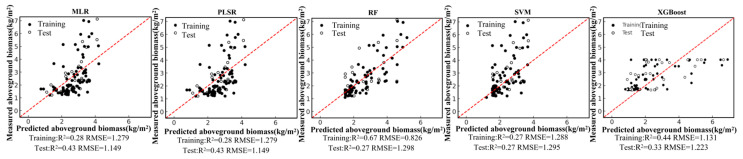
Model fitting performance for NDVI using five machine-learning algorithms.

**Figure 5:**
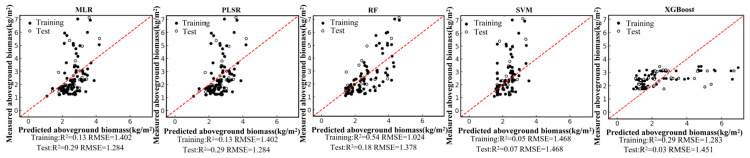
Model fitting performance for PVI using five machine-learning algorithms.

**Figure 6:**
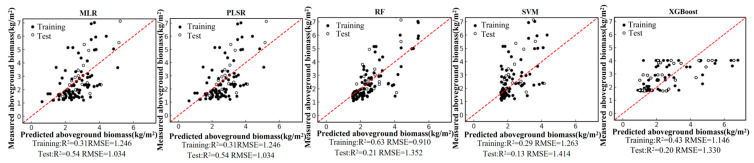
Model fitting performance for B342 using five machine-learning algorithms.

**Figure 7:**
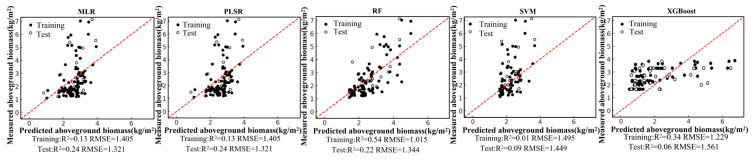
Model fitting performance for B12/34 using five machine-learning algorithms.

**Figure 8:**
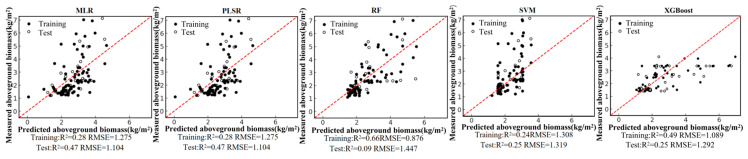
Model fitting performance for EVI using five machine-learning algorithms.

**Figure 9:**
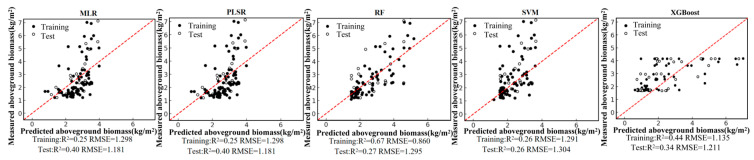
Model fitting performance for MSAVI using five machine-learning algorithms.

**Figure 10:**
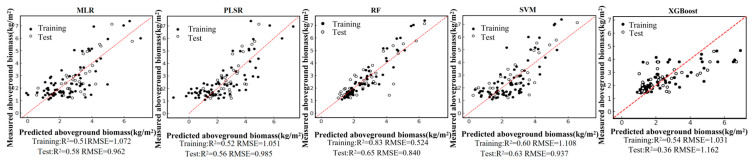
Model fitting performance of the multi-predictor model using five machine-learning algorithms.

Based on the field-measured aboveground biomass (AGB) dataset and the Pearson correlation analysis, six remote-sensing predictors were selected from the candidate variables, including four vegetation indices (NDVI, PVI, EVI, and MSAVI) and two-band ratios (B342 and B12/34). Using these predictors, we developed five regression models—random forest (RF), multiple linear regression (MLR), support vector regression (SVR), partial least squares regression (PLSR), and XGBoost—under a single-predictor (single-variable) setting for each remote-sensing variable. We further constructed multi-predictor models by combining the selected variables to enable a systematic comparison of retrieval accuracy between single-predictor and multi-predictor scenarios across different algorithms. Model performance was evaluated using the coefficient of determination (R2) and root mean square error (RMSE). The single-predictor AGB retrieval results for the six variables are shown in Figure 4, Figure 5, Figure 6, Figure 7, Figure 8 and Figure 9, and the multi-predictor model results are presented in Figure 10. The red dashed line denotes the 1:1 relationship between measured and predicted aboveground biomass (AGB). Solid and open circles represent training and independent test samples, respectively (see legend). Model accuracy is summarized using R^2^ and RMSE (kg m^−2^) for both the training and test sets).

Scatter plots for the five regression models under each remote-sensing predictor scenario indicate that single-index-driven models generally exhibit limited predictive capability. For both linear methods (MLR and PLSR) and non-linear approaches (RF, SVR, and XGBoost), training and testing samples were widely dispersed, with pronounced departures from the 1:1 reference line in the high-AGB range, indicating poor tracking of gradients at larger biomass values. Quantitatively, under single-index scenarios, training R2 typically ranged from 0.10 to 0.40, testing R2 was mostly below 0.35, and RMSE remained approximately 1.2–1.5 kg/m^2^. These results suggest that a single spectral index is insufficient to represent the multidimensional structural properties of roadside vegetation and its non-linear responses to environmental controls, thereby constraining AGB retrieval accuracy in highway corridor settings.

In contrast, model performance improved markedly under the multi-predictor scenario. For all algorithms, the scatter clouds converged toward the 1:1 reference line, indicating reduced systematic bias. The RF model showed the clearest advantage, achieving a training R2 of 0.83, a testing R2 of 0.65, and an RMSE of 0.84 kg/m^2^. This performance indicates that RF can effectively exploit complementary information among vegetation indices and capture complex non-linear relationships. Under the multi-index setting, testing R2 increased to about 0.63 for SVR and about 0.36 for XGBoost, reflecting varying degrees of improvement relative to the single-index scenario. MLR and PLSR also benefited from multi-predictor inputs, with testing R2 rising to 0.56–0.58 and RMSE decreasing accordingly. Overall, expanding from single-index to multi-index predictors substantially strengthened the ability of all models to represent spatial variability in AGB. RF delivered the best generalization performance, and non-linear approaches generally outperformed linear methods. These results suggest that ensemble-learning models driven by multiple vegetation indices are more suitable as a primary strategy for AGB retrieval in highway roadside vegetation systems.

### 3.3. GF-2-Based AGB Mapping and Estimation

**Figure 11:**
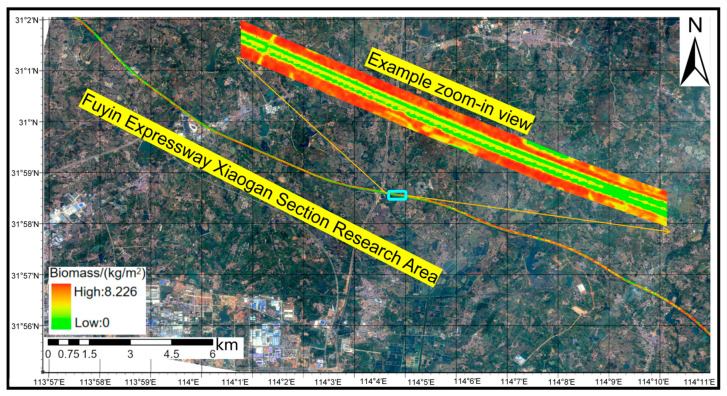
Spatial distribution of aboveground biomass (AGB) within a 30 m roadside buffer along a section of the Xiaogan segment of the Fuyin Expressway. Note: The AGB map represents point estimates; pixel-level uncertainty mapping is discussed in Section 4. The evaluated area (yellow boundary) represents the corridor defined as ±30 m from the dual carriageway centerline, including the paved road width.

Based on the integrated comparison of regression models under the single- and multi-predictor scenarios, the multi-predictor RF model exhibited clear advantages over the other approaches in both fitting accuracy and generalization performance. It was therefore selected as the final model for AGB retrieval in the highway roadside corridor and was used to generate the spatial distribution map for the Xiaogan segment of the Fuyin Expressway (Figure 11). The model was applied on a pixel-wise basis to the 32 km highway section and a 30 m roadside buffer on each side, resulting in an estimated total aboveground biomass of 566.966 t for the study corridor.

**Figure 12:**
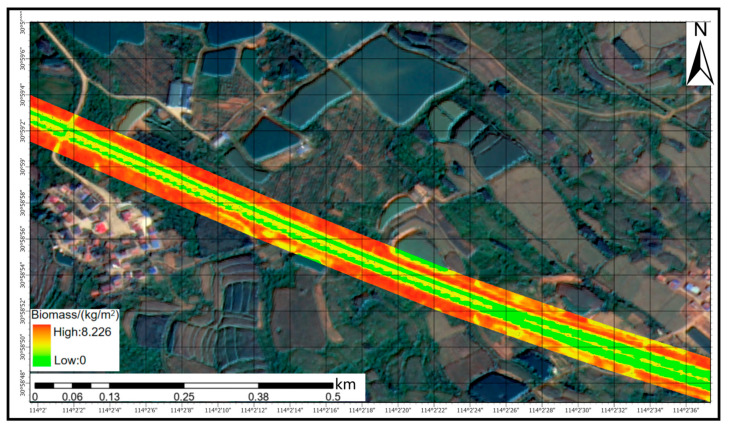
A zoomed-in view of the area outlined by the blue rectangle in Figure 11. Note: This panel is a local zoom-in overlay of the RF-predicted AGB map on the 0.8 m GF-2 pan-sharpened true-colour imagery, intended to facilitate cartographic interpretation of fine-scale features and the corresponding biomass patterns.

As shown in Figure 11 and Figure 12, high-AGB zones form continuous belt-like patterns along both sides of the highway, corresponding to areas dominated by trees or mixed stands, such as shelterbelts beyond the shoulder margins. In contrast, the central median between carriageways is primarily covered by shrubs and low-stature vegetation, yielding generally low-to-moderate AGB estimates and a spatial pattern characterized by discontinuous mid-value strips. The retrieval results also capture localized AGB variations associated with topographic relief, surrounding land-use types, and vegetation configuration: higher AGB values tend to occur near slope toes or in segments with denser vegetation cover, whereas lower values correspond to hardened surfaces or exposed ground. Overall, the mapped AGB pattern is highly consistent with the topographic and landscape context of the highway corridor, indicating that the RF-based retrieval model provides not only high numerical accuracy but also spatially coherent and interpretable estimates.

## 4. Discussion

In linear roadside ecosystems, AGB retrieval from optical imagery is constrained by strong spatial heterogeneity, narrow vegetation belts, and frequent adjacency to non-vegetated surfaces (pavement, bare soil, and water) [30]. In this study, combining complementary spectral predictors (vegetation indices plus band-ratio features) improved AGB estimation compared with single-index inputs, indicating that no single index can fully represent the coupled effects of canopy condition, soil background, and boundary mixing in highway corridors [31]. Across the evaluated algorithms, ensemble learning (especially RF) showed the most stable generalization, consistent with its ability to model non-linear relationships and interactions among predictors while remaining relatively robust to noise and collinearity [32]. The remaining underestimation at high AGB levels is consistent with known limitations of optical vegetation metrics, including index saturation and mixed-pixel effects at vegetation–non-vegetation boundaries, which are amplified in narrow, fragmented targets [33].

Placing our results in the context of prior work, optical AGB mapping studies using medium-resolution sensors (e.g., Sentinel-2 or Landsat) commonly report moderate performance that varies with vegetation type, calibration data, and model choice, and they frequently emphasize the impact of index saturation and background mixing on predictive accuracy. For example, studies using Sentinel-2 with ensemble methods (random forest or gradient boosting) demonstrate that non-linear ensembles can outperform simpler approaches but remain sensitive to training data coverage and landscape heterogeneity, particularly when predictors are primarily optical indices [34]. In addition, spectral reflectance saturation in Landsat imagery and related impacts on forest AGB estimation have been explicitly documented, highlighting that purely optical predictors can lose sensitivity at higher biomass levels [35].

With respect to sensor choice, very high spatial resolution imagery is particularly advantageous for linear roadside vegetation because the target features are narrow, fragmented, and strongly affected by adjacency (road surface, shoulders, ponds, and exposed soil). A 10–30 m pixel (Sentinel-2/Landsat) can be comparable to, or larger than, the width of many roadside greenbelts, increasing mixed-pixel contamination and potentially smoothing corridor-scale heterogeneity, whereas 0.8 m GF-2 data can delineate strips and boundaries more precisely. This advantage is also consistent with recent evidence that integrating GF-2 with medium-resolution imagery can reduce overestimation associated with low-resolution pixels in sparse or fragmented tree targets [35]. Although we did not conduct a cross-sensor experiment in the present study (i.e., training and testing the same models using Sentinel-2/Landsat for the same corridor), the above considerations and published findings support the rationale for selecting GF-2 as the primary data source for corridor-scale AGB retrieval in this work.

In the context of satellite data selection, it is important to clarify the advantages of using GF-2 imagery relative to medium-resolution sensors such as Sentinel-2 (10 m) and Landsat (30 m), particularly for linear roadside vegetation systems. Roadside greenbelts are typically narrow, fragmented, and spatially heterogeneous, making them especially susceptible to mixed-pixel effects when medium-resolution imagery is used. Spectral mixing between vegetation, road surfaces, and surrounding land covers can reduce sensitivity to biomass variability and obscure corridor-scale heterogeneity.

By contrast, the very high spatial resolution of GF-2 (0.8 m) enables more precise delineation of roadside vegetation strips and reduces contamination from adjacent non-vegetated surfaces, which is critical for capturing fine-scale biomass patterns along highway corridors. Although this study did not conduct a direct cross-sensor model comparison, the GF-2-based results highlight the practical advantages of very high spatial resolution imagery for AGB retrieval in fragmented or linear landscapes where spatial detail is a primary constraint.

Accurate estimation of aboveground biomass in roadside and linear infrastructure ecosystems is therefore essential for informing climate-change mitigation strategies and supporting sustainable land-use and transport-infrastructure management [5,36].

From a policy and management perspective, our findings provide a scientific basis for incorporating roadside vegetation into carbon accounting, ecological compensation schemes, and green infrastructure planning. Mapping the spatial distribution and heterogeneity of aboveground biomass offers spatially explicit evidence to guide environmental management and conservation planning, enabling authorities to identify areas with high carbon-storage potential and implement differentiated management strategies.

Despite the overall robustness of the modelling framework, several limitations should be acknowledged. First, tree aboveground biomass (AGB) was estimated using published allometric equations developed for subtropical regions, without local calibration based on destructive sampling in the study area. Although these equations are widely applied and suitable for regional-scale assessments, differences in local species composition, stand structure, and site conditions may introduce uncertainty in absolute AGB estimates. Therefore, the results should be interpreted as regionally representative patterns rather than precise plot-level values.

Second, the number and spatial distribution of field plots were limited and primarily constrained to linear highway corridors. This sampling configuration may reduce the representativeness of training data for other landscape contexts and restrict the extrapolation of the results beyond similar roadside vegetation environments.

Third, potential spatial autocorrelation among nearby roadside plots was not explicitly accounted for in the random training–testing split, which may result in optimistic estimates of model generalization performance. Because roadside plots are spatially clustered along the corridor, nearby samples may share similar environmental and management conditions, violating the independence assumption of random splitting. Therefore, future work should adopt spatially explicit validation strategies (e.g., spatial blocking or spatial cross-validation) to reduce spatial leakage between training and testing sets and to provide a more conservative assess generalization. Such spatial validation designs are particularly important for linear ecosystems where spatial dependence is strong.

In addition, although GF-2 imagery provides high spatial resolution, mixed-pixel effects may still occur at boundaries between vegetation and adjacent road surfaces, particularly in narrow roadside greenbelts, introducing additional uncertainty at the pixel scalein the mapped products. The mapped AGB results are presented as point estimates, and we did not generate a pixel-level uncertainty (or confidence-interval) map in the current version. Uncertainty in corridor-scale AGB mapping may arise from multiple sources, including the use of non-locally calibrated allometric equations, limited and spatially clustered field samples, spatial dependence, and mixed pixels at vegetation–road boundaries. Future work will quantify and map prediction uncertainty using ensemble/bootstrapping-based approaches (e.g., repeated resampling and prediction) or quantile-based methods to provide confidence intervals for management interpretation.

The mapped AGB results are presented as point estimates, and we did not generate a pixel-level uncertainty (or confidence-interval) map in the current version. Uncertainty in corridor-scale AGB mapping may arise from multiple sources, including the use of non-locally calibrated allometric equations, limited and spatially clustered field samples, spatial dependence, and mixed pixels at vegetation–road boundaries. Future work will quantify and map prediction uncertainty using ensemble/bootstrapping-based approaches (e.g., repeated resampling and prediction) or quantile-based methods to provide confidence intervals for management interpretation. At a spatial resolution of 0.8 m, the corridor width corresponds to more than 100 pixels across each transect, which is sufficient to resolve fine-scale roadside vegetation patterns, although mixed pixels may still occur at vegetation–road boundaries.

Future research should expand the sample size and optimize the spatial sampling design to improve training-data coverage and uncertainty control, while integrating complementary data sources such as hyperspectral and/or LiDAR observations to enhance sensitivity to vegetation spectral-structural characteristics. In addition, modelling and validation strategies capable of identifying or controlling spatial autocorrelation—such as spatial blocking or spatial cross-validation—should be employed to avoid inflated generalization performance caused by spatial dependence, thereby improving model robustness and transferability [37,38]. Furthermore, linking ecological-monitoring outcomes with socio-economic dimensions (e.g., cost–benefit trade-offs, governance constraints, and human well-being) can generate more actionable management insights and enhance the framework’s policy relevance. As linear green infrastructures along transport corridors, roadside vegetation belts can support biodiversity and provide multiple ecosystem services, while also being subject to practical constraints related to safety, maintenance, and operational requirements of roadway management. Therefore, planning and management of roadside greenbelts should balance ecological benefits with safety regulations, maintenance costs, and adjacent land-use constraints. Integrating ecosystem-service indicators into corridor-scale risk and vulnerability assessments can support more targeted strategies for climate adaptation and sustainable transport-corridor and landscape management [5].

## 5. Conclusions

(1) Using field-measured AGB samples and GF-2 high-resolution imagery, this study targeted highway roadside corridors as a representative linear ecosystem. It evaluated four vegetation indices (NDVI, PVI, EVI, and MSAVI) and two-band ratios (B3/4/2 and B12/34) for AGB retrieval. Through correlation analysis and redundancy diagnostics, we constructed a representative and complementary multi-index feature set, providing a reproducible framework for variable selection in fine-scale remote-sensing monitoring of roadside vegetation.
(2) We compared five machine-learning models (MLR, PLSR, SVR, RF, and XGBoost) under single-index and multi-index feature scenarios. Single-index models showed limited capacity to represent AGB spatial heterogeneity in highway corridors, whereas performance improved substantially under the multi-index setting SVR. RF achieved the best overall performance, with training R^2^ of about 0.83, testing R^2^ of about 0.65, and RMSE of about 0.84 kg/m^2^, highlighting the advantage of ensemble learning in exploiting multi-source spectral information and modelling complex non-linear relationships within linear roadside vegetation environments.
(3) Applying the optimal multi-index RF model, we mapped AGB for a 32 km section of the Xiaogan segment of the Fuyin Expressway with a 30 m roadside buffer on both sides. High AGB values were mainly distributed along tree-dominated belts beside the highway, while the central median and hardened shoulder areas exhibited comparatively lower values. The total AGB within the study corridor was estimated at approximately 566.97 t, which should be interpreted as a regionally representative estimate rather than a precise plot-level value, suggesting substantial biomass stock and associated carbon sequestration potential. Overall, integrating high-resolution optical remote sensing, multi-index feature combinations, and ensemble-learning methods provides a practical framework for AGB retrieval in linear roadside vegetation systems and can support roadside greening management, ecological monitoring, and corridor-scale carbon assessment for transport infrastructure.

## Figures and Tables

**Table 1:** A summary of the source of receipt, spatial resolution, and acquisition conditions of all initial datasets.

Dataset	Source of Receipt	Spatial Resolution (m)	Conditions of Receipt
GF-2 multispectral imagery (Blue, Green, Red, NIR)	China Centre for Resources Satellite Data and Application (CRESDA)	3.2	Acquired in the growing season (June–July); cloud cover < 5%; Level-1A products; radiometrically and geometrically corrected
GF-2 panchromatic imagery	China Centre for Resources Satellite Data and Application (CRESDA)	0.8	Acquired simultaneously with multispectral imagery; used for pan-sharpening
Ground AGB sample plots	Field measurements by the authors	Plot level (1 × 1, 2 × 2, 5 × 5, 10 × 10)	Measured during the same growing season as satellite acquisition; biomass calculated using species-specific allometric equations
Road vector data	Local transportation authority/OpenStreetMap	-	Used to define highway centerlines and extract roadside vegetation belts

**Table 2:** Species-specific allometric equations used to estimate aboveground biomass (AGB).

Vegetation Type	Biomass Component	Equation Form	Parameters	Source
*Cinnamomum camphora*	Whole tree	W = a·DBH^b^	a = 0.2191, b = 2.0052	[16]
*Populus* spp.	Aboveground	W = a·DBH^b^	a = 0.0955, b = 2.4284	[16]
*Populus* spp.	Aboveground	W = a·DBH^b^·H^c^	a = 0.0584, b = 2.0519, c = 0.5916	[16]
Soft broadleaf group	Stem	W = a·DBH^b^	a = 0.0440, b = 1.7095	[17]
Soft broadleaf group	Root	W = a·DBH^b^	a = 0.0417, b = 2.0247	[17]
Hard broadleaf group	Stem	W = a·DBH^b^	a = 0.0560, b = 1.8140	[17]
Hard broadleaf group	Root	W = a·DBH^b^	a = 0.0549, b = 2.0953	[17]
Shrub	Whole plant	W = (0.2652 + 0.0367·DBH^2^·H)·N	-	[18]

Note: DBH is diameter at breast height (cm), H is tree height (m), and N is the number of shrub individuals within each plot. All equations estimate dry biomass (kg per individual). When species-specific equations for Hubei Province were unavailable, equations developed for subtropical regions of China were adopted due to comparable climatic conditions and vegetation composition.

**Table 3:** Description of GF-2 bands.

Band Type	Band Name	Wavelength Range (nm)	Spatial Resolution
Panchromatic (PAN)	Pan	450–900	0.8 m
Multispectral (MS)	Blue	450–520	3.2 m
Multispectral (MS)	Green	520–590	3.2 m
Multispectral (MS)	Red	630–690	3.2 m
Multispectral (MS)	NearInfrared (NIR)	770–890	3.2 m

**Table 4:** Formulas of vegetation indices and band-ratio predictors derived from GF-2 bands.

Predictor	Formula
Two-band ratios	$B_{12}=B_{1}/B_{2},B_{13}=B_{1}/B_{3},B_{14}=B_{1}/B_{4},B_{23}=B_{2}/B_{3},$ $B_{24}=B_{2}/B_{4},B_{34}=B_{3}/B_{4}$
Three-band ratio combinations	$B_{123}=(B_{1}+B_{2}){/B}_{3},\;B_{124}=(B_{1}+B_{2}){/B}_{4},B_{132}=(B_{3}+B_{1})/B_{2}$, $B_{134}=(B_{1}+B_{3})/B_{4},\;B_{142}=(B_{1}+B_{4})/B_{2},\;B_{143}=(B_{1}+B_{4})/B_{3}$, $B_{231}=(B_{2}+B_{3})/B_{1},\;B_{234}=(B_{2}+B_{3})/B_{4},\;B_{241}=(B_{2}+B_{4})/B_{1},$ $B_{243}=(B_{4}+B_{2})/B_{3},\;B_{341}=(B_{3}+B_{4})/B_{1},\;B_{342}=(B_{3}+B_{4})/B_{2}$
Four-band ratio combinations	$B_{12/34}=(B_{1}+B_{2})/(B_{3}+B_{4}),\;B_{13/24}=(B_{1}+B_{3})/(B_{2}+B_{4}),$ $B_{14/23}=(B_{1}+B_{4})/(B_{3}+B_{2}),\;B_{23/14}=(B_{3}+B_{2})/(B_{1}+B_{4}),$ $B_{123/4}=(B_{1}+B_{2}+B_{3})/B_{4},\;B_{234/1}=(B_{2}+B_{3}+B_{4})/B_{1}$
Vegetation indices	$\mathrm{NDVI}=\frac{B_{4}-B_{3}}{B_{4}+B_{3}},\;\mathrm{DVI}={\;B}_{4}-B_{3},\;\mathrm{RVI}=\frac{B_{4}}{B_{3}},\;\mathrm{EVI}=2.5\frac{\left(B_{4}-B_{3}\right)}{B_{4}+6B_{3}-7B_{1}+1},$ $\mathrm{SAVI}=(1+0.5)\frac{B_{4}-B_{3}}{B_{4}+B_{3}+0.5},\;\mathrm{ARVI}=\frac{B_{4}-({2B}_{3}-B_{1})}{B_{4}+({2B}_{3}-B_{1})}$

Note: In this table, B_1_, B_2_, B_3_, and B_4_ represent the digital number (DN) values of the blue, green, red, and near-infrared (NIR) bands of the GF-2 imagery, respectively.

**Table 5:** Pearson correlation analysis between candidate predictors and aboveground biomass (AGB).

**Factor**	**B1**	**B2**	**B3**	**B4**	**B12**	**B13**	**B14**	**B23**	**B24**
Correlation coefficient	−0.249 **	−0.350 **	−0.486 **	0.430 **	0.424 **	0.49 **	−0.361 **	0.501 **	−0.590 **
**Factor**	**B34**	**B123**	**B124**	**B134**	**B142**	**B143**	**B231**	**B234**	**B241**
Correlation coefficient	−0.542 **	0.300 *	−0.489 **	−0.489 **	0.638 **	0.621 **	−0.451 *	−0.573 **	0.534 **
**Factor**	**B243**	**B341**	**B342**	**NDVI**	**SAVI**	**RVI**	**DVI**	**EVI**	**PVI**
Correlation coefficient	0.600 **	0.280 **	0.493 *	0.672 **	0.672 **	0.670 **	0.651 **	0.640 **	0.650 **
**Factor**	**MSAVI**	**GNDVI**	**B234/1**	**B134/2**	**B124/3**	**B123/4**	**B12/34**	**B13/24**	**B14/23**
Correlation coefficient	0.688 **	0.645 **	0.516 **	0.608 **	0.629 **	−0.574 **	−0.540 **	−0.602 **	0.574 **

Note: ** indicates an extremely significant correlation at the 0.01 level (two-tailed), and * indicates a significant correlation at the 0.05 level (two-tailed).

## Data Availability

The datasets and material used during this study are available from the corresponding authors upon reasonable request.

## Supplementary Materials

The following supporting information can be downloaded at: https://www.mdpi.com/article/10.3390/s26051729/s1, Figure S1: Random forest-based recursive feature elimination (RF-RFE) process for predictor selection.
