# Supplementary figures and images for: Unlocking Roadside Carbon Sequestration Potential: Machine Learning Estimation of AGB in Highway Vegetation Belts Using GF-2 High-Resolution Imagery
Source: Sensors (Basel). 2026 Mar 9;26(5):1729. doi: 10.3390/s26051729 (PMC12987276; doi:10.3390/s26051729)

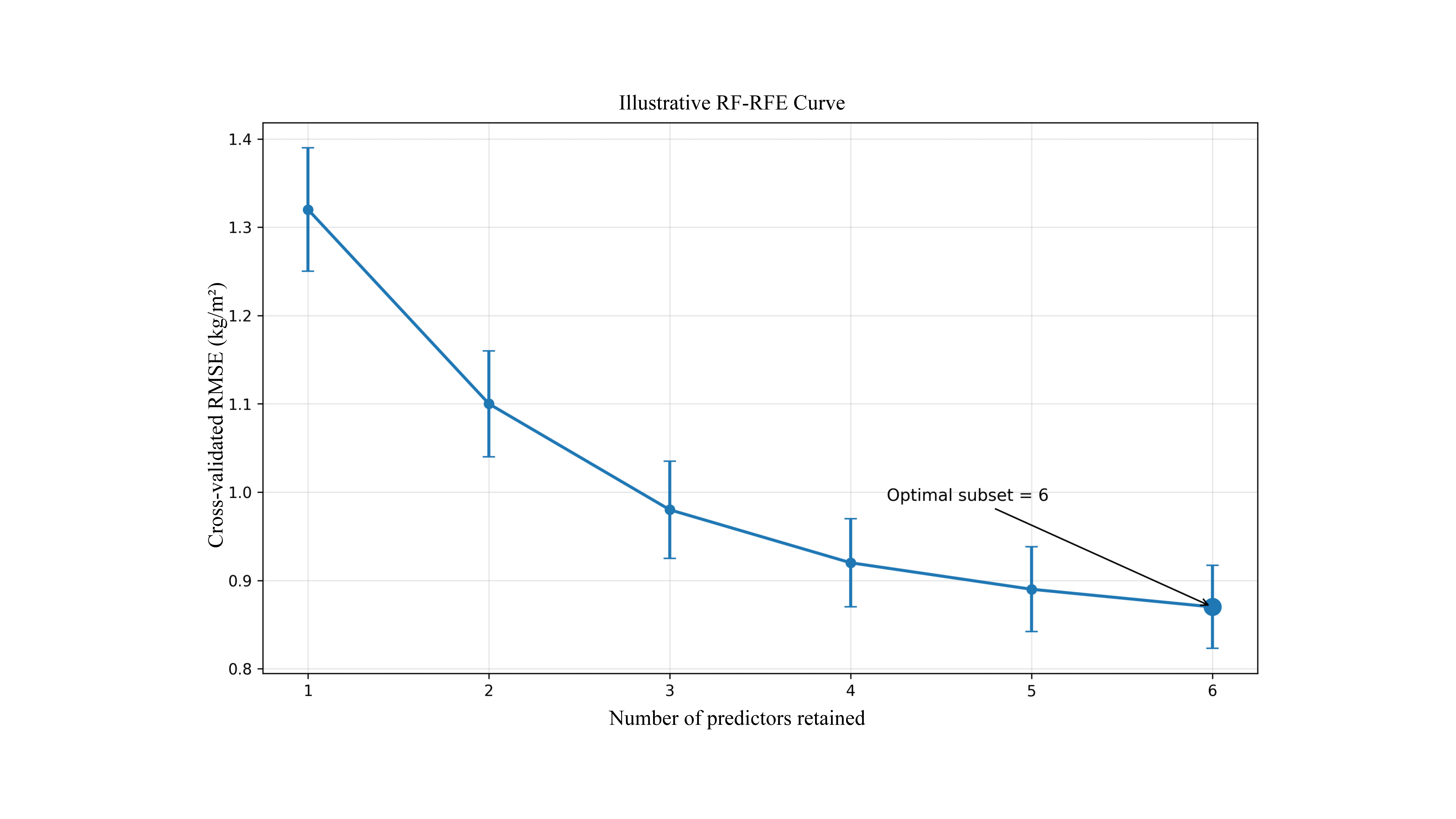

Supplement: Supplementary file 1 [file sensors-26-01729-s001.zip › sensors-4098095-supplementary.png]
